# TNS1 and TNS4 play a potential role in development of pancreatic ductal adenocarcinoma but not TNS2 and TNS3

**DOI:** 10.1080/19336918.2026.2650871

**Published:** 2026-04-01

**Authors:** Natalia Świsłocka, Anna Pryczynicz, Justyna Dorf, Konrad Zaręba, Katarzyna Guzińska-Ustymowicz

**Affiliations:** a Department of General Pathomorphology, Medical University of Bialystok, Bialystok, Poland; b Department of Clinical Laboratory Diagnostics, Medical University of Bialystok, Bialystok, Poland; c Department of Oncological Surgery and General Surgery, Independent Public Healthcare Center of the Ministry of the Interior and Administration in Bialystok, Bialystok, Poland

**Keywords:** Adhesion proteins, carcinogenesis, immunohistochemistry, intraductal secretion, local progression, pancreatic cancer, Tensins

## Abstract

Tensins are a family of adhesion proteins that play a role in constructing the cytoskeleton, as well as in intracellular and extracellular communication. Their expression was evaluated in 22 pancreatic cancer patients using the immunohistochemistry method. TNS1 expression occurred more frequently among patients with tumor diameter ≥ 2 cm, which may suggest an association with the development of pancreatic cancer. Intraductal TNS1 was observed less often with presence of necrosis and hemorrhages in tumor. The fact that cancer cells secrete TNS1 suggests that it could be investigated as a potential target for liquid biopsies. TNS4 expression occurred more frequently among females and was observed when necrosis in tumor was strong. TNS2 and TNS3 are not involved in the development of ductal pancreatic adenocarcinoma.

## Introduction

Pancreatic cancer represents a significant clinical problem due to its severe and rapid course, lack of targeted therapy and very high mortality rate. A small number of cases of pancreatic cancer are operable at the time of detection; in addition, relatively early detected pancreatic cancers infiltrate adjacent tissues and induce a strong local response in the form of fibrosis (desmoplastic reaction). It mostly affects older individuals (70 years of age), and the greatest environmental influence (doubling the risk of the disease) is smoking. The disease entities most predisposing to the development of cancer are chronic pancreatitis and diabetes. The most common histological type of pancreatic cancer is ductal adenocarcinoma of intermediate to poor differentiation, most of cases formed in the head of the pancreas. In half of the cases, tumors located in the head of the pancreas obstruct the distal common bile duct, giving symptoms of jaundice. Regardless of the primary location, pancreatic cancers often invade the extraperitoneal space, with a tendency to infiltrate around nerves [1]. According to WHO statistics, the incidence and mortality of pancreatic cancer is increasing [2]. In Poland, it was the 9th most common cancer in terms of incidence among men in 2022. Additionally, pancreatic cancer ranked fifth in terms of mortality in women and sixth in men, respectively [3].

Adhesion proteins may play a role in the carcinogenesis and progression of pancreatic cancer. One group of adhesion proteins are Tensins. This is a small family of proteins that includes: Tensin-1, Tensin-2, Tensin-3 and Tensin-4 (TNS1, TNS2, TNS3 and TNS4). They are an important structural element of cells, as they are involved in the formation of focal adhesion bridges, connecting the intracellular actin cytoskeleton to beta-integrins (responsible for trans-membrane connections). In this way, they ensure the integrity of the cytoskeleton and condition the possibility of its reorganization. They are also involved in the formation of signaling pathways between the intracellular matrix and the extracellular matrix. Further functions of Tensins, which may play a very important role in carcinogenesis, include being responsible for the process of cell migration, cell proliferation and cell apoptosis – the oncogenic potential depends on these factors [4,5].

The premise of research on the expression of Tensins in malignant tumors is to discover targets for possible therapies that could improve patients’ quality of life and (to a greater or lesser extent) enhance treatment effectiveness. Tensins, as a family of proteins involved in the construction of the cytoskeleton and focal adhesion site, perform their function in processes such as migration, proliferation and the interaction between the interior of cells and the extracellular matrix. These functions are responsible for tumor progression. Therefore, the aim of this study was to investigate the relationship between the immunohistochemical expression of Tensins in histopathological sections of pancreatic cancer and the clinicopathological parameters of this tumor.

## Materials and methods

### Study group

The study was conducted on a group of 22 patients (11 males and 11 females, aged 23–86) diagnosed with pancreatic ductal carcinoma, treated in the 2nd Clinical Department of General and Gastroenterological Surgery of the University Teaching Hospital of Bialystok between 2005 and 2017. Tissue material was borrowed from the archives of the Academic Centre for Pathomorphological and Genetic-Molecular Diagnostics in Bialystok. Patients were qualified for the study group after applying the following criteria: diagnosed pancreatic ductal carcinoma at any stage. Exclusion criteria included: pancreatic intraepithelial neoplasia (PanIN), malignant neoplasm of endocrine pancreas, and other non-epithelial neoplasms, metastasis of other neoplasms to the pancreas and lack of complete medical records. TNS1, TNS2, TNS3 and TNS4 expressions were assessed in tissue samples by immunohistochemistry. Clinicopathological parameters were analyzed in correlation with protein expression.

### Tissue material

Tissue sections collected during surgery were fixed in 4% buffered formalin and embedded in paraffin. The paraffin cubes were sliced on a microtome into slices of approx. 4 µm, and were then stained with hematoxylin and eosin. In a routine histopathological examination, the histological type of tumor, grade of malignancy (G), T stage (pT), lymph node metastases (pN), foci of hemorrhage within the tumor, inflammatory infiltration, degree of necrosis and degree of desmoplasia were assessed. The following information was selected from the histopathological diagnosis documents: age and sex of the patients, diameter and location of the tumor, absence or presence of distant metastases. The study was conducted in accordance with the Declaration of Helsinki and authorized by the Bioethics Committee of the Medical University of Bialystok, no.: APK.002.126.2024. Written consent for the scientific research to be conducted on their tissue material was provided by each participant in the study.

### Immunohistochemical staining

Immunohistochemical (IHC) staining was performed on 22 pancreatic cancer tissues using a polymer method. Paraffin blocks were cut into slices of approx. 4 µm on a microtome on silanised slides. Tissue specimens were incubated at 60°C overnight in an incubator. They were then deparaffinised in a series of xylene and hydrated by passing through a series of alcohols of decreasing concentration (2x99.9%, 96%, 70%). Tissue sections were heated in a water bath in citrate buffer pH = 6.0 at 97.5°C for 20 minutes to expose the antigen and then cooled at room temperature for 20 minutes. After blocking endogenous peroxidase for 10 minutes with 3% hydrogen peroxide, Protein Block (ImmPRESS HRP Universal Antibody (Horse Anti-Mouse/Rabbit IgG) Polymer Detection Kit, Peroxidase; Vector Laboratories; Catalog #: MP-7500–15, Germany; 100 μl per slide) was applied to prevent nonspecific antibody binding. The next step was incubation with polyclonal anti-Tensin-1 antibody (Anti-TNS1 rabbit polyclonal antibody, Sigma-Aldrich, Catalog #: HPA036089, Sweden; 100 μl per slide), diluted 1:50; anti-Tensin-2 antibody (Anti-TNS2 rabbit polyclonal antibody Sigma-Aldrich, Catalog #: HPA034659, Sweden; 100 μl per slide), diluted 1:200; anti-Tensin-3 antibody (Anti-TNS3 rabbit polyclonal antibody, Sigma-Aldrich, Catalog #: HPA056015, Sweden; 100 μl per slide), diluted 1:100 and anti-Tensin-4 antibody (Anti-TNS4 rabbit polyclonal antibody, Biorbyt, Catalog #: orb186458, United Kingdom; 100 μl per slide) diluted 1:50 for 30 minutes at room temperature. The ImmPRESS Universal Antibody Polymer Reagent kit (ImmPRESS HRP Universal Antibody (Horse Anti-Mouse/Rabbit IgG) Polymer Detection Kit, Peroxidase; Vector Laboratories; Catalog #: MP-7500–15, Germany; 100 μl per slide; 100 μl per slide) and ImmPact DAB chromogen (ImmPACT® DAB Substrate, Peroxidase (HRP), Vector Laboratories, Catalog #: SK-4105, Germany; 100 μl per slide) were used to visualize the reaction. Cell nuclei were stained with hematoxylin. Specimens were then dehydrated in a series of alcohols of increasing concentration and washed in xylene solutions [6].

### Validation of the detection of TNS1, TNS2, TNS3 and TNS4 expression

We focused on ensuring precise results from the immunohistochemical staining procedure. In order to achieve satisfactory results during the TNS1, TNS2, TNS3 and TNS4 protein staining procedure, positive and negative controls were performed, selected dilutions of primary antibodies (1:50, 1:100, 1:200) and selected incubation times (30 minutes, 60 minutes) were tested. Normal pancreatic tissue from around the tumor was used as a negative control. In contrast, tissue specimens of normal gastric, renal and colorectal tissues, i.e. tissues recommended by the antibody manufacturer, in which the expression of individual Tensins is highest, were used as positive controls [6].

### Microscopic evaluation

The specimens were viewed under an Olympus B×41 light microscope and assessed by two independent pathomorphologists. The expression of TNS1, TNS2, TNS3 and TNS4 proteins was assessed under 100x magnification in 10 representative fields of view. In each field of view, ≥100 tumor cells were assessed. TNS1 protein expression was observed both in the cytoplasm of the cells and in the lumen of the ducts of the exocrine portion of the tumor tissue, while TNS4 expression was observed only in the cytoplasm of the tumor cells. The presence of TNS1 protein in the cytoplasm of >5% of tumor cells was considered positive expression. Exocrine expression of TNS1 was assessed separately and described as present or absent. Cytoplasmic expression of TNS4 protein in >10% of tumor cells was considered positive. No TNS2 and TNS3 expression was observed in pancreatic cancer cells [6].

### Statistical analysis

Clinicopathological parameters (age, sex, location, tumor diameter, histological grade, TNM staging, absence or presence and grade of necrosis, hemorrhagic foci, inflammatory infiltration and desmoplasia) were analyzed in correlation with TNS1 (both cytoplasmic and secretory) and TNS4 protein expression. Comparison of the expression of both proteins was performed using a Student’s t-test Comparison of TNS1 and TNS4 protein expression with selected clinicopathological parameters was performed using the Man-Whitney U test for two groups and the Kruskal-Wallis test for three or more groups. Additionally, Spearman’s correlation coefficient test was performed to demonstrate the correlation between the studied parameters. *p* < .05 value was considered statistically significant. Statistica 13.3 software (Statsoft, Cracow, Poland) was used for the analysis. Missing data were removed in pairs.

## Results

### Expression of TNS1, TNS2, TNS3 and TNS4 proteins in normal pancreatic tissue compared with cancer

**Figure 1.**
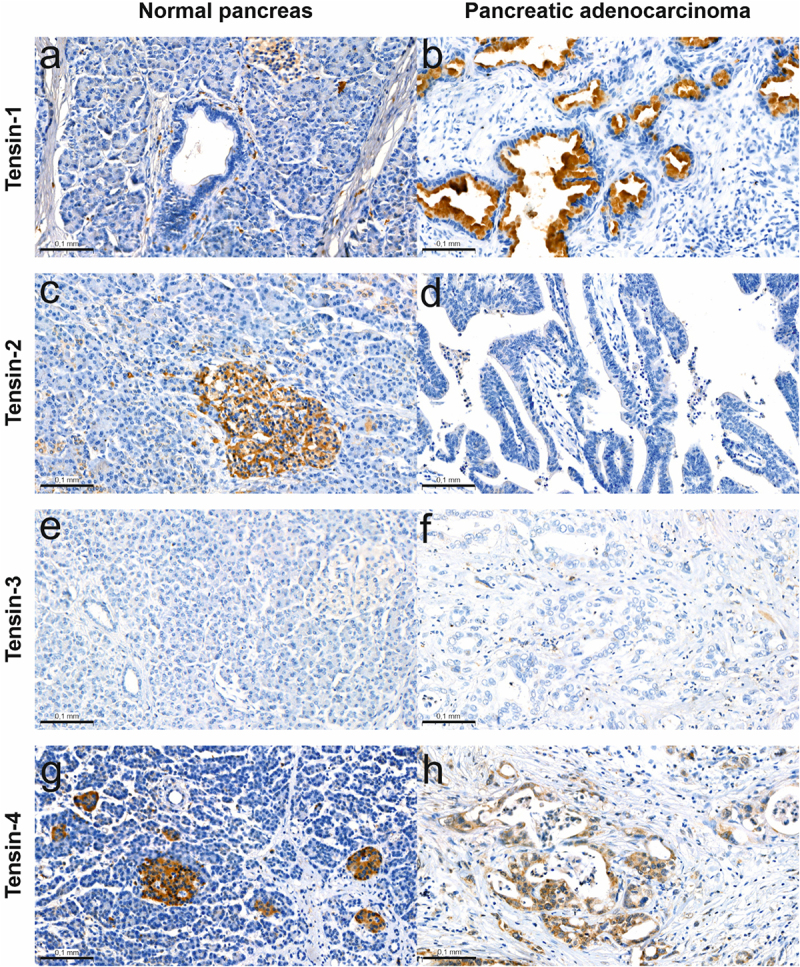
Representative immunohistochemical stainings of Tensins in normal and cancer tissue of pancreas. TNS1 protein expression in normal pancreas – weak expression in islets of Langerhans and no expression in pancreatic ducts (a), and pancreatic cancer – cytoplasmic expression in pancreatic duct cancer cells and intraductal secretion (b). TNS2 protein expression in normal pancreas – strong expression in islets of Langerhans and no expression in pancreatic ducts (c), and pancreatic cancer – no expression in pancreatic duct cancer cells (d). TNS3 protein expression in normal pancreas – lack of expression in islets of Langerhans and lack of expression in pancreatic ducts (e), and pancreatic cancer – lack of expression in pancreatic duct cancer cells (f). TNS4 protein expression in normal pancreas – strong expression in islets of Langerhans and no expression in pancreatic ducts (g), and pancreatic cancer – cytoplasmic expression in pancreatic duct cancer cells (h).

In the normal pancreatic tissue, weak expression of TNS1 and strong expression of TNS2 and TNS4 was observed in the pancreatic islets of Langerhans, while no expression of the above Tensins was observed in the exocrine part of the pancreas or in the pancreatic ducts. In the case of TNS3 protein, a complete lack of expression was noted in the normal pancreatic tissue. TNS1 expression was observed in cancer cells’ cytoplasm and secreted into cancer glands in 15/22 cases (68.2%). TNS4 expression was observed in cancer cells’ cytoplasm in 7/22 (31.82%) cases. TNS2 and TNS3 did not show expression in the tumor tissue in any case of pancreatic cancer (Figure 1).

### Comparison of TNS1 and TNS4 protein expression with clinicopathological parameters of pancreatic cancer

Statistical analysis did not reveal any significant differences between TNS1 expression (cytoplasmic and secreted) and gender, age or histological differentiation degree. There was no correlation with additional histopathological parameters such as desmoplasia and inflammatory cell infiltration. However, a positive correlation was observed between the tumor diameter, presence of necrosis and its degree of advancement and presence of hemorrhage foci and its degree of advancement. TNS1 intraductal secretion occurred more frequently among patients with tumor diameter ≥ 2 cm (81.2%) (*p* = .023). The expression of intraductal TNS1 was observed less often with presence of necrosis in tumor and its medium and strong advancement level (20% and 0%, respectively) (*p* = .042). The secretion of TNS1 in cancerous ducts decreases in cases which presented hemorrhages in tumor (33.3%) (*p* = .024). In contrast, the cytoplasmic expression of TNS1 showed statistical significance only with tumor diameter, where the presence of expression correlated with tumor size ≥ 2 cm (*p* = .048) (Table 1). A statistical significant correlation was observed between the TNS4 cytoplasm expression and gender of a patient. The cytoplasmic TNS4 expression in cancer cells occurred more frequently among females vs males (54.6% and 9.1%, respectively, *p* = .021). Moreover, the cytoplasmic expression of TNS4 was observed when necrosis in tumor was strongly present (*p* = .014). Although, no expression was observed when necrosis was weak or moderate. There were no statistically significant correlations in reference to other clinicopathological parameters (Table 2).

**Table 1.** Correlations between immunohistochemical expression of TNS1 in cytoplasm and secreted TNS1 in tumor tissue and clinicopathological parameters of pancreatic cancer. Missing data were removed in pairs.

Parameters	TNS1 cytoplasmic expression		*p* value	TNS1 secreted expression		*p* value
	Absent N (%)	Present N (%)		Absent N (%)	Present N (%)	
**Age** <60 ≥60	2 (50%) 5 (29.4%)	2 (50%) 12 (70.6%)	.476	2 (50%) 5 (29.4%)	2 (50%) 12 (7.6%)	.476
**Sex** Male Female	4 (36.4%) 4 (36.4%)	7 (63.6%) 7 (63.6%)	1.000	2 (18.2%) 5 (45.5%)	9 (81.8%) 6 (54.5%)	.193
**Localisation** Head Body Tail Diffuse lesion	5 (45.5%) 0 (0%) 1 (33.3%) 1 (25%)	6 (54.5%) 3 (100%) 2 (66.7%) 3 (75%)	.524	3 (27.3%) 1 (33.3%) 1 (33.3%) 1 (25%)	8 (72.7%) 2 (66.7%) 2 (66.7%) 3 (75%)	.992
**Tumor diameter** <2 cm ≥2 cm	2 (100%) 4 (25.%)	0 (0%) 12 (75%)	.**048**	2 (100%) 3 (18.8%)	0 (0%) 13 (81.2%)	.**023**
**Grade of malignancy** Medium differentiated Poorly differentiated	7 (35%) 1 (50%)	13 (65%) 1 (50%)	.732	6 (30%) 1 (50%)	14 (70%) 1 (50%)	.620
**T stage** 1–2 cm or less 2 - between 2 cm and 4 cm 3 - larger than 4 cm but is still within the pancreas	1 (100%) 1 (12.5%) 6 (60%)	0 (0%) 7 (87.5%) 4 (40%)	.071	1 (100%) 3 (37.5%) 3 (30%)	0 (0%) 5 (62.5%) 7 (70%)	.403
**Lymph node metastasis** Absent Present	3 (25%) 5 (50%)	9 (75%) 5 (50%)	.251	5 (41.7%) 2 (20%)	7 (58.3%) 8 (80%)	.307
**Distant metastasis** Absent Presen	8 (38.1%) 0 (0%)	13 (61.9%) 1 (100%)	1.000	7 (33.3%) 0 (0%)	14 (66.7%) 1 (100%)	1.000
**Necrosis** Absent Weak Moderate Strong	0 (0%) 3 (37.5%) 3 (60%) 2 (66.7%)	6 (100%) 5 (62.5%) 2 (40%) 1 (33.3%)	.134	1 (16.7%) 1 (12.5%) 2 (40%) 3 (100%)	5 (83.3%) 7 (87.5%) 3 (60%) 0 (0%)	.042*
**Foci of haemorrhage** Absent Single Numerous	4 (36.4%) 0 (0%) 4 (57.1%)	7 (63.6%) 4 (100%) 3 (42.9%)	.180	1 (9.1%) 1 (25%) 5 (71.4%)	10 (9.9%) 3 (75%) 2 (28.6%)	.**024**
**Inflammatory infiltration** Weak Absent Medium Strong	1 (100%) 1 (14.3%) 3 (33.3%) 3 (60%)	0 (0%) 6 (85.7%) 6 (66.7%) 2 (40%)	.234	1 (100%) 1 (14.3%) 3 (33.3%) 2 (40%)	0 (0%) 6 (85.7%) 6 (66.7%) 3 (60%)	.369
**Desmoplasia** Poor Prominent	1 (16.7%) 7 (43.8%)	5 (83.3%) 9 (56.2%)	.269	2 (33.3%) 5 (31.2%)	4 (66.7%) 11 (68.8%)	.963

*The expression of intraductal TNS1 decreases with presence and increasing level of advancement of the necrosis in tumor (Spearman’s correlation coefficient test; *p* = .016, Coefficient = −0.504).

**Table 2.** Correlations between immunohistochemical expression of TNS4 in cytoplasm in tumor tissue and clinicopathological parameters of pancreatic cancer. Missing data were removed in pairs.

Parameters	Tensin-4 expression		*p* value
	Absent N (%)	Present N (%)	
**Age** <60 ≥60	4 (100%) 10 (58.8%)	0 (0%) 7 (41.2%)	.127
**Sex** Male Female	10 (9.9%) 5 (45.5%)	1 (9.1%) 6 (54.5%)	.**021**
**Localisation** Head Body Tail Diffuse lesion	8 (72.7%) 2 (66.7%) 2 (66.7%) 2 (50%)	3 (27.3%) 1 (33.3%) 1 (33.3%) 2 (50%)	.885
**Tumor diameter** <2 cm ≥2 cm	2 (100%) 12 (75.0%)	0 (0%) 4 (25.0%)	.452
**Grade of malignancy** Medium differentiated Poorly differentiated	14 (70%) 1 (50%)	6 (30%) 1 (50%)	.584
**T stage** 1–2 cm or less 2 - between 2 cm and 4 cm 3 - larger than 4 cm but is still within the pancreas	1 (100%) 6 (75%) 5 (50%)	0 (0%) 2 (25%) 5 (50%)	.424
**Lymph node metastasis** Absent Present	7 (58.3%) 8 (80%)	5 (41.7%) 2 (20%)	.299
**Distant metastasis** Absent Presen**t**	15 (71.4%) 0 (0%)	6 (28.6%) 1 (100%)	.147
**Necrosis** Absent Weak Moderate Strong	3 (50%) 7 (87.5%) 5 (100%) 0 (0%)	3 (50%) 1 (12.5%) 0 (0%) 3 (100%)	.**014**
**Foci of haemorrhage** Absent Single Numerous	10 (9.9%) 2 (50%) 3 (42.9%)	1 (9.1%) 2 (50%) 4 (57.1%)	.079
**Inflammatory infiltration**Absent Weak Medium Strong	1 (100%) 3 (42.9%) 7 (77.8%) 4 (80%)	0 (0%) 4 (57.1%) 2 (22.2%) 1 (20%)	.377
**Desmoplasia** Poor Prominent	4 (66.7%) 11 (68.8%)	2 (33.3%) 5 (31.2%)	.929

## Discussion

Tensin expression under physiological as well as pathological conditions influences cell biological activity. The most important function of Tensins is the co-formation of focal connections that mediate the regulation of biological processes in cells in response to extracellular and intracellular stimuli. Participation in signal transduction is closely linked to the regulation of cell motility and growth dynamics, which may promote tumorigenesis and metastasis. However, it should be mentioned that cell migration in various tumor types is associated with decreased expression or overexpression of Tensins [7].

To date, there are no reports in the literature on the expression of Tensins in tumor tissue of ductal pancreatic cancer and the correlation with clinicopathological parameters of this cancer. This is the first report of its kind, so for comparison purposes we have compared our results with those for other widely studied tumors.

**Figure 2.**
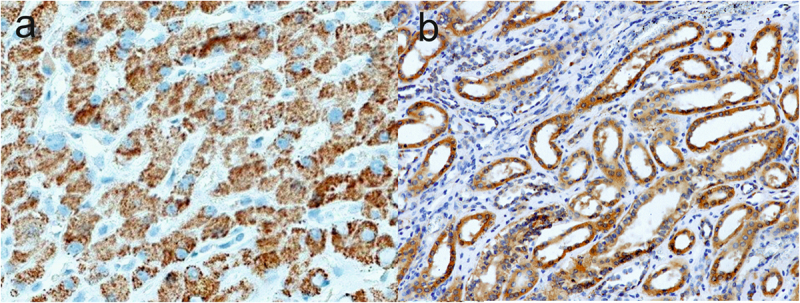
Positive IHC staining for TNS2 and TNS3 proteins in tissue controls: (a) positive expression of TNS2 in normal hepatocytes and (b) positive expression of TNS3 in renal tubules.

In our study, TNS1 showed expression in the cancerous tissue in more than two-thirds of patients. There are no literature reports on TNS1 expression in pancreatic cancer, but expression was observed in gastric cancers in 7.78% of cases. It was also observed that in gastric cancer, TNS1 was overexpressed in peritoneal metastatic lesions compared to primary tumor tissue [8]. In our study, TNS4 protein showed expression in 30% of patients with pancreatic cancer, whereas in other studies conducted on pancreatic cancer, expression was observed in as many as 70.45% of patients [9]. In other cancers, positive TNS4 expression is observed in approximately 55–72% of patients with gastric cancer [10–12], in 90% of patients with breast cancer [13], in 50.7% of patients with esophageal squamous cell carcinoma [14], in 52.1% of patients with lung cancer [15], in 43%-55% of patients with hepatocellular carcinoma [16,17], in 41%, and 46% of patients with primary melanoma and metastatic melanoma, respectively [18]. Different results are observed for the other Tensins, in our study TNS2 and TNS3 did not show expression in the tumor tissue in any patient, while in the study by Cheng LC et al [19], strong expression of TNS2 was demonstrated in 100% of pancreatic cancer specimens. However, the researchers used a different clone of the antibody (for isoforms 2 and 3). In gastric cancer, TNS2 and TNS3 showed 4.44% and 35.56% of expression, respectively [7], TNS2 in GIST was observed in 71.4% of patients [20], and TNS3 in renal cell carcinoma in 59% of patients [21]. These discrepancies in results may indicate the specificity of Tensin expression in cancers and other malignancies. The correct selection of the antibody clone is important, due to the fact that individual Tensins occur in different tissues at different intensities and may have more than one isoform, e.g. like TNS2. Due to the differences in our negative TNS2 and TNS3 staining results in PDAC with other studies, the correctness of our staining is evidenced by the positive expression of these proteins in control tissues (Figure 2).

With regard to the histological features of pancreatic cancer, we did not note a correlation between Tensin expression and histopathological grade, whereas the following correlations were observed in the course of other cancers: high TNS1 expression in early-stage lung adenocarcinoma compared with late-stage [22], in gastric cancer, an increase in TNS4 expression was observed more frequently in tumors without a mucosal component compared with mucinous carcinomas and TNS4 expression was higher in moderately differentiated tumors than in poorly differentiated and undifferentiated tumors [10], in breast cancer, TNS4 expression was significantly associated with higher histological grade [13]. The above reports indicate the specificity of the influence of Tensin expression in individual cancers on their histological features.

Considering the role of Tensins in cell motility, there are reports in the literature on its involvement in tumor progression. Our study did not show a correlation between Tensin expression and TNM staging; however, the relationship of increased expression of both cytoplasmic and endocrine TNS1 and larger tumor diameter was significant. This may indicate the participation of TNS1 in the local growth process of pancreatic cancer. Studies comparing TNS1 expression and pancreatic cancer staging are lacking, but in gastric adenocarcinomas an effect of TNS1 expression on tumor staging was observed-expression was more frequently present in tumors with distant metastases present [6]. In contrast, studies in breast cancer show the opposite results – a decrease in TNS1 expression with the presence of metastases to adjacent lymph nodes [23]. In other cancers, correlations between the expression of individual Tensins and tumor progression have also been found. In GIST, strong TNS2 staining was associated with the absence of metastases [20]. In kidney cancer, TNM staging and tumor size were negatively correlated with TNS3 plasma membrane staining in the tumor [21]. TNS4 remains the most studied Tensin of the family, and its expression in other cancers was indicative of higher tumor grade and higher TNM staging. In esophageal squamous cell carcinoma, positive TNS4 expression was significantly associated with the presence of lymph node metastases [14], and in colorectal cancer, high TNS4 expression was associated with the presence of distant metastases [24], in breast cancer, TNS4 expression was significantly associated with larger primary tumor size and axillary lymph node involvement [13], in primary melanoma, significantly stronger TNS4 staining was noted in tumors ≥ 1 mm thick [18] and in hepatocellular cancer, TNS4 expression was positively correlated with primary tumor size and TNM stages [17].

The primary limitation of our study was a small cohort of patients. Statistical significances could therefore be a coincidence as a consequence of the limited number of cases. To enhance the reliability of the research results we provided, the experiment needs to be conducted with a greater number of patients. Furthermore, our study was limited by the lack of data on survival in pancreatic cancer patients. There are also no such reports in the available literature and, given some confirmed role of Tensins in pancreatic cancer, such analyses should be performed. In other cancers, patient survival may be associated with Tensin expression. A correlation was shown, in which higher TNS4 expression was associated with shorter survival. Significantly poorer prognosis, shorter survival time and metastasis-free survival was correlated with high TNS4 expression in breast cancer patients [13], in gastric cancer patients [11], in esophageal squamous cell carcinoma patients [14], and in colorectal cancer patients [24]. In melanoma, strong TNS4 expression was significantly associated with worse 5-year overall and disease-specific survival rates in patients [18], and in hepatocellular cancer overall survival time and five-year recurrence-free survival time were significantly shorter in patients with high TNS4 expression compared with patients with low TNS4 expression [17]. For TNS1, an inverse relationship was observed. In breast cancer, high TNS1 expression promoted prolonged metastasis-free survival [23], while in clear-cell renal-cell carcinoma, the presence of membrane staining of TNS3 provided a statistically significant survival advantage for patients over tumors with cytoplasmic expression alone [21].

In addition, a correlation between Tensin expression and other clinical and histopathological parameters specific to individual tumors has been observed. However, these are isolated reports in the literature. In our study, a significant association was observed between the presence of intraductal secretion of TNS1 and larger tumor size, intraductal secretion of TNS1 was observed less often with presence of necrosis in tumor and its medium and strong level of advancement, and it was noted that secretion of TNS1 in cancerous ducts decreases in cases which presented hemorrhages in tumor. The literature reports the importance of protein markers secreted into the lumen of the pancreatic ducts, later detected in pancreatic juice and blood plasma in the course of PDAC. This is due to the fact that intraductal pancreatic juice secretion is in direct contact with the ductal epithelial lining from which PDAC arises [24,25]. In addition, there was noticed an association between cytoplasmic expression of TNS4 and female sex and the complete absence of necrosis and its strong presence. It is worth mentioning that these significances may be biased due to the small study group. We see a need to repeat the experiment on a larger number of patients to increase the credibility of the research results that we presented. The role of TNS1 and TNS4 in the course of pancreatic ductal cancer is therefore unclear, and no similar reports have been found in the scientific literature. Studies comparing immunohistochemical expression of Tensins and other clinicopathological parameters have been performed on other cancers, including those originating in the gastrointestinal tract. In gastric cancer according to Lauren classification, increased TNS4 expression was observed in the intestinal type [10], high TNS4 expression in colorectal cancer patients was associated with advanced Dukes stage [26], in breast cancer TNS4 expression was significantly associated with poor Nottingham Prognostic Index [13], in hepatocellular cancer TNS4 expression was positively correlated with venous invasion [19]. Strong TNS4 expression was observed in 35% of patients with melanomas in AJCC stage I compared to 47% of patients with melanomas in AJCC stages II – IV [18]. In GIST, strong TNS2 staining was associated with location (stomach), female gender and tumors of lower risk categories [20].

## Conclusion

The presence of TNS1 in ductal pancreatic cancer may suggest an association between its expression and the development of cancer cells. This is also confirmed by increased TNS1 expression as the tumor grows. Changes in expression of TNS1 do not affect the stage or histological type of pancreatic cancer. The fact that cancer cells secrete TNS1 invites future investigation into its potential as a liquid biopsy target. Female gender and its’ constitution of the system (metabolism, hormones, composition) may predispose to expression of TNS4 by pancreatic ductal carcinoma tumor cells. Based on scientific articles and preliminary observation, it is warranted to assess further study of the role of TNS4 in modulating the necrosis of cancer cells. It may be the subject of further research aimed at examining its exact function in promoting cancer development. TNS4 May also be a biomarker for various cancers, including pancreatic ductal carcinoma. Lack of statistical significance indicates that TNS2 and TNS3 are not involved in tumorigenesis and do not influence the course of ductal pancreatic adenocarcinoma development.

## Data Availability

Data available on request due to privacy/ethical restrictions.
